# Rhubarb Anthraquinones Protect Rats against Mercuric Chloride (HgCl_2_)-Induced Acute Renal Failure

**DOI:** 10.3390/molecules21030298

**Published:** 2016-03-08

**Authors:** Dan Gao, Ling-Na Zeng, Pin Zhang, Zhi-Jie Ma, Rui-Sheng Li, Yan-Ling Zhao, Ya-Ming Zhang, Yu-Min Guo, Ming Niu, Zhao-Fang Bai, Xiao-He Xiao, Wei-Wei Gao, Jia-Bo Wang

**Affiliations:** 1 Institute of Medicinal Plant Development, Chinese Academy of Medical Sciences, Beijing 100094, China; gaodan_gd@163.com; 2 China Military Institute of Chinese Medicine, 302 Military Hospital, Beijing 100039, China; zeng_lingna85@126.com (L.-N.Z.); zhaoyl_123@126.com (Y.-L.Z.); zymprchina@126.com (Y.-M.Z.); guoyuming_123@126.com (Y.-M.G.); niumingbright@126.com (M.N.); bai_zf001@126.com (Z.-F.B.); 3 Integrative Medicine Center, 302 Military Hospital, Beijing 100039, China; zhang_ping77@126.com (P.Z.); pharmacy302xxh@126.com (X.-H.X.); 4 Beijing Friendship Hospital Attached of Capital Medical University, Beijing 100050, China; mazhij_123@126.com; 5 Experimental Animal Center, 302 Military Hospital, Beijing 100039, China; ruisheng_sci@126.com

**Keywords:** mercury, acute renal failure, rhubarb, anthraquinones, protection

## Abstract

Mercury (Hg) causes severe nephrotoxicity in subjects with excess exposure. This work attempted to identify whether a natural medicine—rhubarb—has protective effects against mercuric chloride (HgCl_2_)-induced acute renal failure (ARF), and which of its components contributed most to the treatment. Total rhubarb extract (TR) were separated to the total anthraquinones (TA), the total tannins (TT) and remaining component extract (RC). Each extract was orally pre-administered to rats for five successive days followed by HgCl_2_ injection to induce kidney injury. Subsequently, renal histopathology and biochemical examinations were performed *in vitro* to evaluate the protective effects. Pharmacological studies showed that TR and TA, but not TT or RC manifested significant protection activity against HgCl_2_-induced ARF. There were also significant declines of serum creatine, urea nitrogen values and increases of total protein albumin levels in TR and TA treated groups compared to HgCl_2_ alone (*p* < 0.05). At last, the major components in TA extract were further identified as anthraquinones by liquid chromatography coupled mass spectroscopy. This study thus provides observational evidences that rhubarb could ameliorate HgCl_2_-induced ARF and its anthraquinones in particular are the effective components responsible for this activity in rhubarb extract.

## 1. Introduction

Mercury (Hg), widely used in many industrial processes, is recognized as a hazardous environmental pollutant and is thought to be poisonous when it enters the body through the respiratory passage, digestive tract, and skin [1,2]. In the early 1900s, the Minamata disease disaster in Japan brought attention to the great danger posed by organic mercury compounds, in this case methyl mercury [3]. The various forms of mercury present different biological behavior, pharmacokinetics, and clinical manifestations [4]. Acute poisoning with mercuric salts, typically mercuric chloride (HgCl_2_), generally targets the gastrointestinal tract and the kidney. Kidney is the primary target organ where mercury is readily accumulated, and expresses its toxicity [5]. Studies have shown that patients with acute mercury poisoning should be given dialysis in acute renal failure. Either single or combined treatment with antidotes, chelating agents including penicillamine, *meso*-2,3-dimercaptosuccinic acid (DMSA) and 2,3-dimercaptopropane-1-sulfonate (DMPS) are often applied to accompany dialysis [6,7]. However, the potential toxicity and side effects such as gastrointestinal discomfort, immunotoxic effects or embryo/fetal toxic effects of synthetic reagents have been a conundrum for a long time [8,9,10]. Thus, researchers have sought to find alternative mercury intoxication treatment methods using natural substances such like herbs with high efficiency and low side effects.

Rhubarb has been widely used as a medicinal herb in China for thousands years. It is stipulated in the Chinese Pharmacopoeia as the dry root and rhizoma of *Rheum palmatum* L., *R. tangguticum* Maxim. ex Balf. or *R. offcinale* Baill, wherein *R. palmatum* L. is the most commonly used [11]. Rhubarb has a variety of functions, including laxative, cholagogic, antibacterial and antineoplastic effects, as well as protective effects to liver and kidney had been reported to inhibit hepatic stellate cell activation or attenuate metabolic disorders [12,13,14]. In recent years, rhubarb has been found to have a curative effect in chronic or acute renal failure (CRF or ARF) in animal models and patients [15,16]. A clinical trial showed that rhubarb is able to reduce proteinuria and improve renal function by itself. It may also have positive cumulative effects when combined with angiotensin-converting enzyme inhibitors [16]. In experimental studies, it was demonstrated that rhubarb could raise the survival rate and improve renal functions of five-sixth nephrectomized ARF mice through inhibiting the pathological development of kidney along with significantly reducing blood serum urea nitrogen (BUN) and creatinine (CREA) [17,18,19].

However, with all of the renal protective potentials described above, nobody has studied the effects of rhubarb in the treatment of mercury-induced renal failure before. Furthermore, considering the diverse ingredients in rhubarb, such as anthraquinones, tannins and polysaccharides [20], the specific effects of each rhubarb active substance associated with renal protection have been inadequately studied. Accordingly, in this paper, to fully dissect the therapeutic potential of rhubarb in mercury-induced renal failure, we implemented molecular studies to identify active ingredients by separating the total extract of rhubarb (TR) into three parts, the total anthraquinones (TA), the total tannins (TT) as well as the remaining components (RC). Then, rigorous pharmacological studies were carried out to analyse the protective effects of each extract against the HgCl_2_-induced ARF in order to provide evidence-based exploration of their mechanism of action. 

## 2. Results 

### 2.1. Common Changes and Survival 

**Figure 1:**
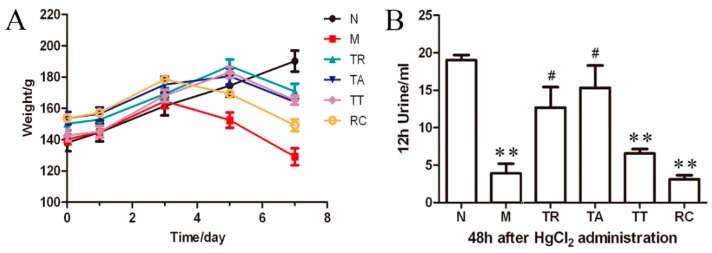
Rhubarb extract protects against body weight and urine volume declines induced by HgCl_2_ injection. (**A**) Comparison of the body weight change of surviving rats in the different groups during the experiment. The body weight of HgCl_2_-injured rat groups stopped increasing and even decreased from the second day after the injection of HgCl_2_. The body weights of the total extract of rhubarb (TR), the total anthraquinones (TA) and the total tannins (TT) groups changed less than those in the HgCl_2_ alone group and the remaining components group (RC); (**B**) Comparison of the 12 h urine volume of surviving rats in the different groups at 48 h after HgCl_2_ injection. The urinary volumes decreased notably after HgCl_2_ intoxication. However, the urinary volumes of rats in the TR and TA groups were non-significantly different to the N group. ** *p* < 0.01 *vs.* group N; ^#^ *p* < 0.05 *vs.* group M.

As expected, HgCl_2_ injection induced emaciation and inactivity in rats compared to the placebo group. However, TR- or TA-treated groups showed significantly less effects compared to other HgCl_2_-treated groups. These improvements included slower decline in body weight (Figure 1A). Compared with the N group, the body weight of HgCl_2_-injured rat groups stopped increasing and even decreased from the second day after the injection of HgCl_2_. The body weights of the TR, TA and TT groups changed less than those in the HgCl_2_ alone group (M) and the RC group. The 12 h urinary volumes at 48 h after HgCl_2_ administration decreased notably after HgCl_2_ intoxication. However, the urinary volumes of rats in the TR and TA groups had almost recovered to normal level and were non-significantly different to the N group. The urinary volumes of rats with TT treatment was slightly higher than that of the M group, while that of the RC group was similar to the M group (Figure 1B). All the TR- and TA-treated rats had a urine color change from yellow to red. Durative diarrhea only appeared in the TA-treated rats. The survival of animals was shown in Table 1. There were only 1/8 rat death in each of the TR and TA groups, while the survival rates were lower than 50% in other HgCl_2_-injured groups.

### 2.2. Histopathology

**Figure 2:**
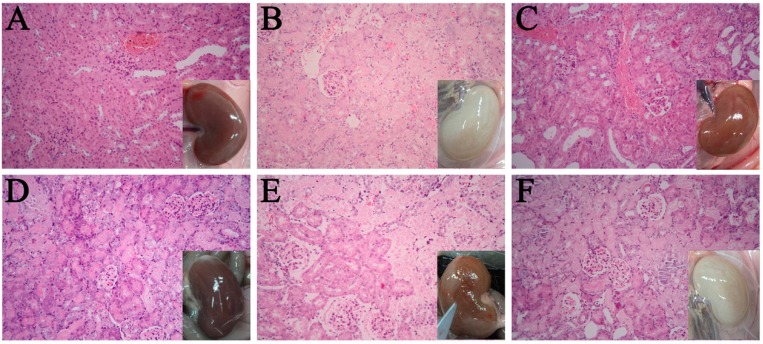
The rats in the total extract of rhubarb and the total anthraquinones groups showed amelioration in renal degeneration from kidney pictures and histopathologic section photos (HE stained, 200× magnification). (**A**) The control group; (**B**) the HgCl_2_ alone group; (**C**) the total extract of rhubarb group; (**D**) the total anthraquinones group; (**E**) the total tannins group and (**F**) the remaining components group.

From the first signs presented in Figure 1, the study was selected for histopathology evaluation. A summary of histopathologic results for the different animal groups is shown in Table 1. The kidney pictures and typical histopathologic section photos are shown in Figure 2. White kidney appearance and jellied enterocoelia exudates were observed in the RC and M groups, suggesting evident histopathologic lesions in their kidneys. In the TT group, ascites were observed and the rat kindeys were yellow and had an enlarged shape. In the TA groups, the color of kidneys was a little yellowish, while the size was similar to those in the N group. Finally, in the TR group, both size and color of the kidneys were similar to normal rats.

After injection of HgCl_2_, the principal histopathologic alterations involving the proximal tubules and glomeruli were characterized from moderate to marked necrosis of tubule epithelial cells in group M. The pattern of renal lesions found in the TT and RC groups were similar to those observed in group M, such as denatured and necrotic epithelia, proximal tubule epithelial cell swelling, granular degeneration with extensive necrosis, and interstitial vascular dilatation and congestion. In contrast, the rats in the TR and TA groups showed amelioration in vacuolar degeneration, lymphocytic infiltration and necrosis in renal tubule epithelial cells (Figure 2). Taken together, the TR and TA, but not TT and RC could rescue kidney’s morphological alterations.

### 2.3. Biochemical Changes 

**Figure 3:**
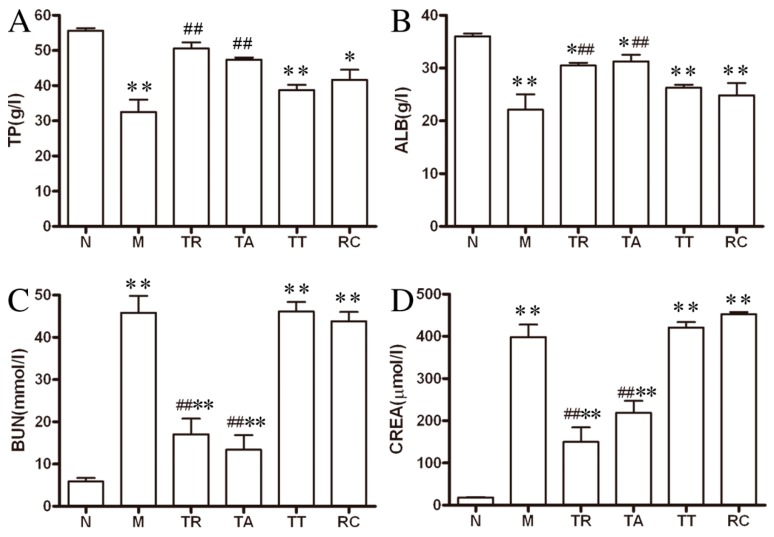
Compared to the HgCl_2_ alone group, blood serum urea nitrogen (BUN) and creatinine (CREA) values of rats were significantly lowered both in the total extract of rhubarb and the total anthraquinones-treated groups (*p* < 0.01) and the level of total protein (TP) and albumin (ALB) clearly increased (*p* < 0.01) at the end of the experiment. (**A**) The level of TP in blood serum; (**B**) the level of ALB in blood serum; (**C**) the level of BUN in blood serum and (**D**) the level of CREA in blood serum. The data were presented as means ± SE of eight rats in each group.* *p* < 0.05, ** *p* < 0.01 *vs.* group N; ^##^ *p* < 0.01 *vs.* group M.

To further analyze the renal function of rats, we next measured the biochemical indices such as CREA, BUN, TP and ALB in all study groups. As Figure 3 indicates, the BUN and CREA values that reflecting renal clearance increased markedly in all HgCl_2_-injured groups (*p* < 0.01) compared to the group N. Compared to group M, these two indices were significantly lowered both in the TR and TA-treated groups (*p* < 0.01), which were close to those in the group N. However, the BUN and CREA values didn’t decrease obviously in the other medication groups (TT and RC).

The proteinuria indices, TP and ALB, clearly decreased (*p* < 0.01) after the injection of HgCl_2_. However, all of the medication groups, especially the TR and TA groups, had improvement in the level of TP and ALB, compared to the M group.

### 2.4. Effect on Oxidative Stress

**Figure 4:**
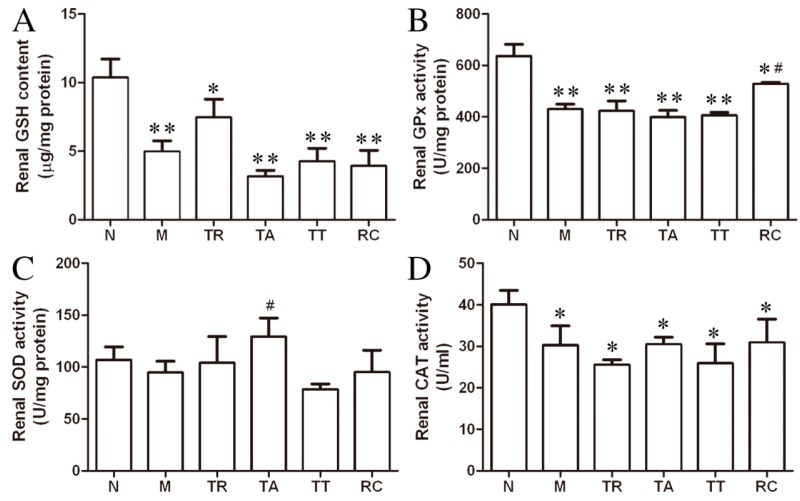
At 48 h after HgCl_2_ administration, there were almost no significant variation observed in superoxide dismutase (SOD) activity, renal catalase (CAT) activity, and renal glutathione (GSH) content of each rhubarb extract group except the remaining components group (RC) for GPx and the total anthraquinones (TA) for SOD activity reversing (*p* < 0.05). (**A**) The level of renal cellular GSH; (**B**) the activity of renal GPx; (**C**) the activity of renal SOD and (**D**) the activity of renal CAT. The data were presented as means + SE of eight rats in each group. * *p* < 0.05, ** *p* < 0.01 *vs.* group N; ^#^ *p* < 0.05 *vs.* group M.

Considering oxidative stress is one of the most dominant detrimental effects of HgCl_2_ intoxication, we measured the changes of key enzymes’ activity with respect to oxidative stress. At 48 h after injection of HgCl_2_, the renal GSH level was almost depleted and the GPx activity decreased significantly, compared to the control group. Compared to the group M, the TR showed a trend of increased (non-significant, *p* > 0.05) intracellular GSH level. However, no effect had been observed in the treatments by individual rhubarb extract (Figure 4A). The HgCl_2_-induced decrease of GPx activity was not reversed by each rhubarb extract treatment except for RC (Figure 4B). Interestingly, the T-SOD and CAT activity in rat kidneys seemed independent of the injection of HgCl_2_ and the treatment of rhubarb as these two indices remained unchanged throughout the whole study.

### 2.5. LC–QTOF-MS Identification for the Components in Each Extract

**Figure 5:**
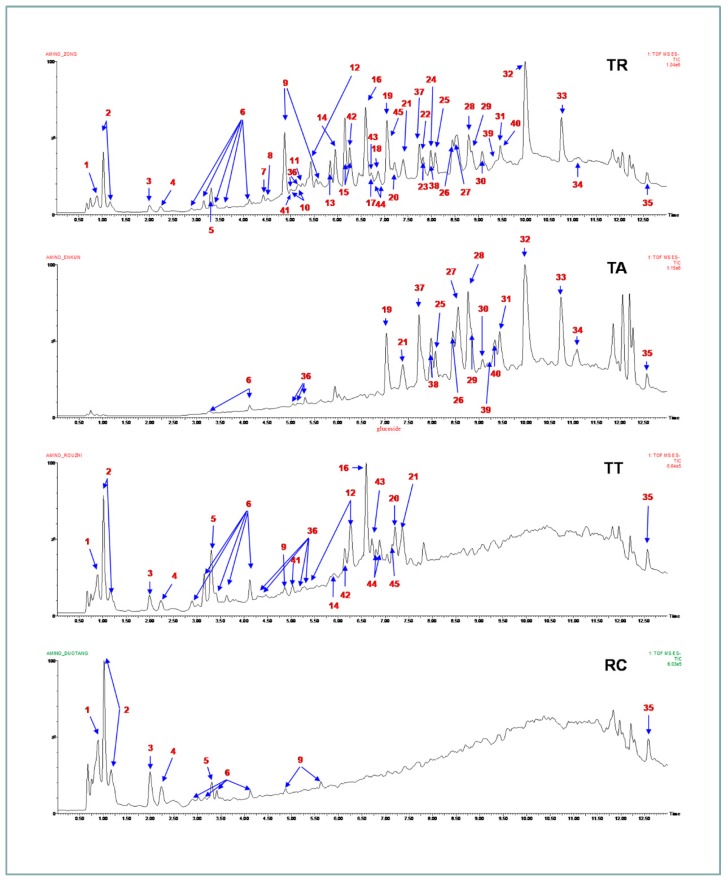
The total ion current chromatograms of each extract were analyzed by HPLC–QTOF-MS in negative ESI mode. Peak numbers are corresponding to compound types in Table 2.

**Figure 6:**
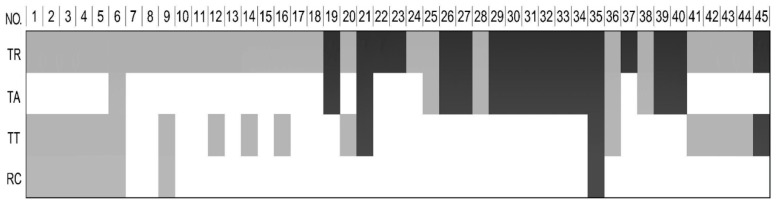
The major constituents of each extract were identified and collectively shown using mass spectrometric data analysis results in Table 2. Colour code: White, not involved; Black, anthraquinones; Gray, non-anthraquinones.

To validate the molecular composition of each rhubarb extract, the optimal LC–QTOF-MS method was applied to each rhubarb extract. The ion current chromatograms of each extract in negative ESI mode are shown in Figure 5, which provided helpful information for confirming molecular weight and structure of the constituents Table 2. A total of 45 compounds have been identified, and 19 of them were found in TA, in which the majority of the intensive peaks were confirmed to be anthraquinone structures identified as follows: rhein, emodin, chrysophanol, physcion and their related glycosides. Among them, five compounds (Nos. 6, 25, 28, 36, 38) were identified as non-anthraquinones and a few (Nos. 6, 21, 35, 36) overlapped with other two extracts (TT and RC) (Figure 6). The data above unambiguously verified that anthraquinones represent the main effective part against ARF in TA.

## 3. Discussion

In our previous study, we have screened some Chinese herbal medicines for potential protective agents against mercury nephrotoxicity. In this paper, we reported a promising hit, *Rheum palmatum* L., commonly called rhubarb and widely used to treat ARF or CRF in China [21]. However, the active constituents in rhubarb against mercury nephrotoxicity remain unclear. Therefore, we separated the total rhubarb extract (TR) into three parts, total anthraquinones extract (TA), total tannins extract (TT) and remaining components extract (RC), and then parallelly investigated the effects of these extracts to HgCl_2_-induced ARF in rats. The results indicated that TR and TA played protective effects against HgCl_2_-induced ARF, while TT and RC had no protection. Moreover, the liquid chromatography coupled mass spectrometry (LC-MS) analysis showed the TA extract was primarily composed of anthraquinones, indicating that the anthraquinones protect rats against mercuric nephrotoxicity.

Through separating TR from rhubarb into TA, TT, RC or keeping it as a whole, we were able to compare their protective effects on mercury intoxication. First of all, all of the three individual fractions or TR did not aggravate renal injury over HgCl_2_; specifically, RC had almost no preventive effect, while TT was low effective and TA had an obvious preventive effect. This was manifested by raising the survival rate, the body weight and the urinary volume of rats. It also improved renal functions through inhibiting the pathological development partly through significantly reducing BUN and CREA values. LC-MS analyses had been used to further determine the detailed molecular composition of each extract. The main mass spectrum peaks in TA spectra were attributed to anthraquinones, which suggests that anthraquinones are the main active ingredients in rhubarb that prevent against HgCl_2_-induced ARF. Further comparisons showed that the efficacy of TA surpassed TR under the comparative doses of crude materials. We speculate that a weakened effect might present in rhubarb due to the coexistence of various components, so it would be reasonable to focus on purified anthraquinones in future studies which might give rise to potential treatment to HgCl_2_-induced ARF. According to our previous research, this anthraquinones part contains a lot of ingredients, notably the rhein, emodin, chrysophanol, physcion, aloe-emodin and their glucosides [21,22], which is consistent with the present study. The anthraquinone glucosides (combined form) are poorly absorbed in intestinal. Instead, those compounds are usually converted by intestinal flora into free anthraquinones that are easier to be absorbed [23]. We consider the free anthraquinones in rhubarb might contribute mostly to the prevention against mercury intoxiction. In addition, despite the fact further investigation is needed, the anthraquinone glucosides might also have effects on the excretion of mercury through the gut due to their laxative activity [24].

Numerous biological and toxicological studies have reported that the nephrotoxicity of mercury or its compounds are mainly caused by the interaction between mercury ions and the nucleophilic site of cellular or subcellular targets. Mercury ion can easily and tightly bind to sulfur, such as thiol group of proteins, peptides and amino acids, which carries mercury ion through sodium ion channels into renal tubule cells, especially in the renal cortex and proximal tubule (S1, S2, S3). Therefore mercury ion could be accumulated gradually which correlate with the severity of renal injury partly through decreased urinary excretion of mercury [5], resulting in severe damage to the renal tubular epithelial cells, lower rate of glomerular filtration and severe oliguria. There are several possible mechanisms that anthraquinones in rhubarb might rescue HgCl_2_-induced renal damage. Firstly, anthraquinones can inhibit renal medulla Na^+^, K^+^-ATP enzyme activity to decrease reabsorption of Na^+^ and discharges increasingly, so as to achieve the diuretic effect [25], thereby contributing to excretion of mercury ion out of body. Furthermore, we suggested that anthraquinones, structurally similar to DMPS or DMSA, can bind to Hg^2+^ directly as a competitive inhibitor to reduce the binding of Hg^2+^ to some sulfur-containing proteins in body [6]. Actually, with the structure of α-phenolic hydroxyl or two adjacent phenolic hydroxyl groups, anthraquinone derivatives have complexation abilities with Hg^2+^ which has been well documented in text books. This interaction was also used as the common procedure to isolate anthraquinones in phytochemistry [26]. Nevertheless, this binding in the physiological conditions *in vivo* needs to be confirmed by further studies. Moreover, anthraquinone constituents such as emodin and aloe-emodin, can inhibit cellular proliferation of various cancer cell, induce apoptosis and prevent metastasis in tumor cells [27]. This injury-attenuation effect may also be one of the detoxification mechanisms of anthraquinone. In addition, it could not be ruled out the possibility mentioned above that anthraquinones have effects on the excretion of mercury through gut due to their laxative activity, even if they might play a small part in the Hg-induced ARF, as HgCl_2_ subcutaneously administered is poorly absorbed from the gastrointestinal tract [28].

Oxidative stress is well documented in the literature to be involved in mercury-induced renal injury [6,28,29]. Indeed, our previous study showed that rhubarb tannins could protect rats from hexavalent chromium induced renal intoxication by scavenging the abundant chromium-induced hydroxyl radical as well as converting hexavalent chromium into non-toxic trivalent chromium, while the anthraquinones did not [30]. However, in this study, we found the anthraquinones has a protective effect on mercury intoxication, while the rhubarb tannins do not. More importantly, our data showed that four kinds of extracts appeared to have little effects on oxidative enzymes activities. The protection mechanism of TA might not be related to antioxidant capacity, suggesting oxidative-stress independent mechanisms. Despite the good antioxidant capacity of tannins [31], there was no obvious therapeutic effect of the rhubarb tannins against HgCl_2_-induced nephrotoxicity. Furthermore, anthraquinones might not only increase the thiol group pool in the cytosol eliminating oxygen radicals and inhibiting lipid peroxidation for the sulfhydryl reactivity, but also offset the imbalance of cellular redox homeostasis for attenuating the depletion in the SH-proteins including some antioxidant enzymes binding on mercury exposure [28,32], suggesting that oxidative stress might not to be involved in the rhubarb treatment of mercury-induced ARF. Deeper analysis is required before any definitive conclusion can be given since Chinese herbal medicines usually manifest their effects by a variety of chemical components which are also multi-linked and multi-targeted in the body.

## 4. Experimental Section

### 4.1. Plant Material and Reagents

The dried root and rhizoma of *Rheum palmatum* L., grown in Lixian County in Gansu Province of China, were purchased from the Lixian Pharmaceuticals Company (Longnan, China) in May 2010. The materials were further identified and validated by Xiao-he Xiao, a taxonomist at the China Military Institute of Chinese Medicine. A voucher specimen (Rh201005Z) was deposited in the institute.

D-101 macroporous resin was supplied by Dajun Technology Development Co., Ltd. (Tianjin, China). Mercuric chloride (HgCl_2_) was obtained from Beijing Chemical Company (Beijing, China). Sodium carboxymethyl cellulose, sodium salicylate, ferric sulphurous acid, hydrogen peroxide and dinitrophenylhydrazine were purchased from the Fuchen Chemical Reagent Factory (Beijing, China). The coomassie brilliant blue kit, glutathione peroxidase (GP_X_) kit, total superoxide dismutase (T-SOD) kit, catalase (CAT) kit and glutathione (GSH) kit were obtained from the Jiancheng Bioengineering Institute (Nanjing, China). GSH was purchased from the Sigma Chemical Co. (St. Louis, MO, USA). 

### 4.2. Preparation and Analysis of Rhubarb Extracts 

The preparation procedures of the rhubarb extracts were explained in detail in our previous paper [30]. In brief, dried rhubarb (1 kg) was extracted repeatedly with 60% ethanol under reflux and then filtered. The eluant was divided into two equal portions. One portion was reserved as the total extract of rhubarb (TR). The other was separated into three parts using D-101 macroporous resin with water and ethanol. These parts were reconfirmed with magnesium acetate methanol solution for the anthraquinones and ferric chloride solution for the tannins according to the color corresponding reactions [33]. Thus, the total anthraquinones (TA) free of tannins, the total tannins (TT) free of anthraquinones, and the remaining components (RC) free of both anthraquinones and tannins were obtained and the yields of TR, TA, TT and RC were 41.2%, 6.73%, 26.1% and 5.92%, respectively. 

The LC-MS analyses were performed on the HPLC instrument (Agilent 1200 series, Waldbronn, Germany) coupled with time-of-flight mass spectrometer (Bruker microTOF-QII, Fremont, CA, USA). The sample was separated on a ZORBAX SB-C18 column (5 μm, 2.1 × 150 mm, Agilent, Santa Clara, CA, USA). The mobile phase consisted of methanol (CH_3_OH) and water, held at 50% CH_3_OH for 5 min, then gradually increased to 95% in 0.1 min, and held at 95% CH_3_OH for another 34.9 min. The flow rate was 0.25 mL/min, and column temperature was set at 25 °C. The diode array detector (DAD) was set at 420 nm. The mass spectrometer parameters were continually optimized in the analysis of the extract so as to obtain relatively high mass responses for the majority of the peaks separated in chromatograms. The optimized parameters in the negative ion mode were as follows: end plate offset −500 V, Funnel 1 RF 200 Vpp, Funnel 2 RF 200 Vpp, capillary 3500 V, dry temperature 180 °C, dry gas 6.0 L/min, ion energy 5 eV. Main compounds were identified by MS and data was analyzed referred to literatures [20,34]. 

### 4.3. Animals and Study Design

Male Sprague-Dawley (S.D.) rats (140–160 g) were procured from the Laboratory Animal Center of the Academy of Military Medical Sciences (Beijing, China, License No.: SCXK 2007-004). The animals were divided into six groups of eight animals each. The control group (N) received placebo treatment with vehicle (0.5% carboxymethylated cellulose in distilled water) throughout the entire experiment. The five model groups were intragastrically administered four rhubarb extracts suspended in vehicle once per day for seven consecutive days. The dose of the TR extract used in this study was set based on preliminary studies in our laboratory. The doses of the other extracts were set according to the yield ratio of each extract from TR to realized comparative doses to each other. The doses of the different extracts were as follows: TR, 1200 mg/kg; TA, 200 mg/kg; TT, 780 mg/kg; and RC, 220 mg/kg. On the fifth day of the experiment, each of the five model groups were subcutaneously administered a single dose of HgCl_2_ (2 mg/kg) aqueous solution to induce acute renal failure in rats. At the same time, the control group received the same volume of pure water subcutaneously. On the eighth day, all the rats were euthanized, and blood samples were collected at room temperature to obtain serum for biochemical determinations. Total urine output over 12 h was collected at 48 h after HgCl_2_ injection over the course of the study. The body weight of each rat was recorded daily. In addition, both kidneys were obtained and weighed, and then the histopathological test was performed. This study was conducted in strict accordance with the recommendations of the Guidelines for the Care and Use of Laboratory Animals of the Ministry of Science and Technology of China. The animal protocol was approved by the Committee on the Ethics of Animal Experiments of the 302 Military Hospital (Approval ID: 11-051).

### 4.4. Histopathological Examination and Biochemical Analysis

For the histopathologic examination, the kidneys were fixed and preserved in 10% neutral buffered formalin, embedded in paraffin, sectioned to a thickness of 5 μm, and stained with hematoxylin and eosin (HE). Serum samples were processed to determine the level of blood serum urea nitrogen (BUN), creatinine (CREA), total protein (TP) and albumin (ALB) using the TBA FR120 automatic biochemistry analyzer (Toshiba Corporation, Tokyo, Japan). 

### 4.5. Measurement of Tissue GSH Level, GPx, T-SOD, and CAT Activities

Kidney tissue was homogenized in cold physiological saline, centrifuged at 3500 rev/min for 10 min at 4 °C, and the supernatant was diluted into different concentrations, that to be assayed for renal cellular GSH level, GPx activity, T-SOD activity and CAT activity according to manufacturer’s instructions. The protein content was determined by Coomassie Brilliant Blue kit. The amount of GSH was expressed as µg/mg protein. GPx activity was then quantified by measuring the rate of change in the absorbance at 412 nm caused by the catalysis of GSH and H_2_O_2_. All of the enzyme activities were expressed as U/mg protein. The activity of T-SOD was determined from the ratio of the auto-oxidation rates measured in the presence or absence of SOD. Reading the changes in absorbance at 405 nm, CAT activity was determined by a catalase assay kit.

### 4.6. Statistics

All results were expressed as the mean ± standard deviation (S.D.). Data were analyzed by one-way ANOVA using the Statistical Package for the Social Sciences of Windows, version 17.0 (SPSS Inc, Chicago, IL, USA). Difference was considered statistically significant when *p* ≤ 0.05, and very significant when *p* ≤ 0.01.

## 5. Conclusions

In summary, this study provided observational evidences that rhubarb plays a protective role against HgCl_2_-induced ARF. Specifically, anthraquinones, *i.e.*, RA extracted and isolated from rhubarb, seem to be the main contributors to the treatment effects rather than tannins. Furthermore, utilizing LC–QTOF-MS, the major components in RA were identified as rhein, emodin, chrysophanol and their glycosides. However, studies of other herbs that are high in anthraquinones are needed to validate whether this protective effect is exclusive to rhubarb. The compounds identified among the anthraquinones could also be further isolated and tested individually to confirm the efficacy and determine whether the observed effects are single or combined effects. Then, hopefully it can be developed as an antidote, chelating pharmaceutical agents in treating HgCl_2_-induced ARF. In fact, this is the first report on the effects of isolated anthraquinones from rhubarb for ARF. It provides a good foundation of further detailed mechanistic studies of anthraquinones on kidney disease treatment. It also opened up great opportunity to develop rhubarb as a resource for potent antidotal, chelating pharmaceutical agents in treating HgCl_2_-induced ARF.

## Figures and Tables

**Table 1:** Survival and pathological alterations by treatment of each rhubarb extract against HgCl_2_-induced acute renal failure.

Groups	Survival ^a^	Renal Tubule Epithelial Cells ^b^			
		Swelling	Granular Degeneration	Necrosis	Interstitial Vascular Congestion
N	8	-	-	-	-
M	2	+++	++++	++++	++++
TR	7	++	+	++	++
TA	7	+	++	+	+
TT	4	++++	+++	+++	+++
RC	3	+++	+++	+++	++++

^a^ The initial number of animals in each group of the study was 8. ^b^ The severity extent of renal tubule epithelial cells necrosis: -, no; +, minimal; ++, mild; +++, moderate; ++++, marked.

**Table 2:** Identifications for the chromatographic peaks.

Peak Number	RT (min)	[M − H]^−^	Identification	Source
1	0.89	195.0505	galactonic acid	TR, TT, RC
2	1.02, 1.16	683.2243	galabiose	TR, TT, RC
3	2.00	191.0189	citric acid	TR, TT, RC
4	2.23	128.0351	pyroglutamic acid	TR, TT, RC
5	3.31	169.0136	gallic acid	TR, TT, RC
6	2.89, 3.16, 3.42, 3.63, 4.13	331.0665	galloyl glucose	TR, TA, TT, RC
7	4.42	577.1337	procyanidin b5	TR
8	4.51	451.1238	catechin glucoside	TR
9	4.88, 5.54	289.0716	catechin	TR, TT, RC
10	5.01, 5.16	729.1448	epicatechin-epicatechin-gallate	TR
11	5.25	635.0876	tri-*O*-galloyl glucose	TR
12	5.33	881.1569	di-*O*-galloyl procyanidin	TR, TT
13	5.84	435.1291	catechin *O*-rhamnopyranoside	TR
14	5.95	441.0816	catechin-*O*-gallate	TR, TT
15	6.15, 6.26	477.1388	coumaroyl-*O*-galloyl glucose	TR
16	6.6	541.1349	resveratrol-(*O*-galloyl)-glucose	TR, TT
17	6.72	629.1499	coumaroyl-di-*O*-galloyl glucose	TR
18	6.86	571.1446	rhapontigenin-(*O*-galloyl)-glucose	TR
19	7.05	445.0756	rhein-*O*-glucoside	TR, TA
20	7.22	393.1177	hydroxymusizin-*O*-glucopyranoside	TR, TT
21	7.4	861.191	sennoside A/B	TR, TA, TT
22	7.74	487.0867	rhein-*O*-acetyl glucose	TR
23	7.82	947.1873	sennoside	TR
24	7.98	623.176	dicoumaroyl-*O*-galloyl glucose	TR
25	8.07	407.1344	torachrysone-*O*-glucoside	TR, TA
26	8.44	415.1017	chrysophanol-*O*-glucoside	TR, TA
27	8.54	431.0966	emodin-*O*-glucoside	TR, TA
28	8.79	607.1818	dicinnamoyl-*O*-galloyl glucose	TR, TA
29	8.86	457.1127	chrysophanol-*O*-(*O*-acetyl)-glucoside	TR, TA
30	9.07	445.1121	rhein-*O*-glucoside	TR, TA
31	9.47	269.0452	aloe-emodin	TR, TA
32	9.99	283.0239	rhein	TR, TA
33	10.76	269.0448	emodin	TR, TA
34	11.11	253.0501	chrysophanol	TR, TA
35	12.6	283.2631	physcion	TR, TA, TT, RC
36	4.26, 4.34, 5.05, 5.14, 5.30	483.0777	di-*O*-galloyl glucose	TR, TA, TT
37	7.72	487.0856	rhein-*O*-(*O*-acetyl)-glucoside	TR, TA
38	7.99	623.1789	dicoumaroyl-*O*-galloyl glucose	TR, TA
39	9.19	473.1071	aloe-emodin-*O*-(*O*-acetyl)-glucoside	TR, TA
40	9.33	487.1239	rhein-*O*-(*O*-acetyl)-glucoside	TR, TA
41	5.03	49.1072	eriodictyol-glucose	TR, TT
42	6.14	7.0456	ethyl gallate	TR, TT
43	6.72	629.1501	coumaroyl-di-*O*-galloyl glucose	TR, TT
44	6.80, 6.87	571.1438	rhapontigenin-*O*-galloyl glucose	TR, TT
45	7.04	847.2088	sennoside C/D	TR, TT
